# Supplementary material for: Opto-p53: A light-controllable activation of p53 signaling pathway
Source: Cell Struct Funct. 2025 Jun 12;50(1):145–56. doi: 10.1247/csf.25017 (PMC12706508; doi:10.1247/csf.25017)
Supplement: Supplementary file 3 — Supplementary Materials [file csf_50_25017_3.pdf]

Supplementary Materials

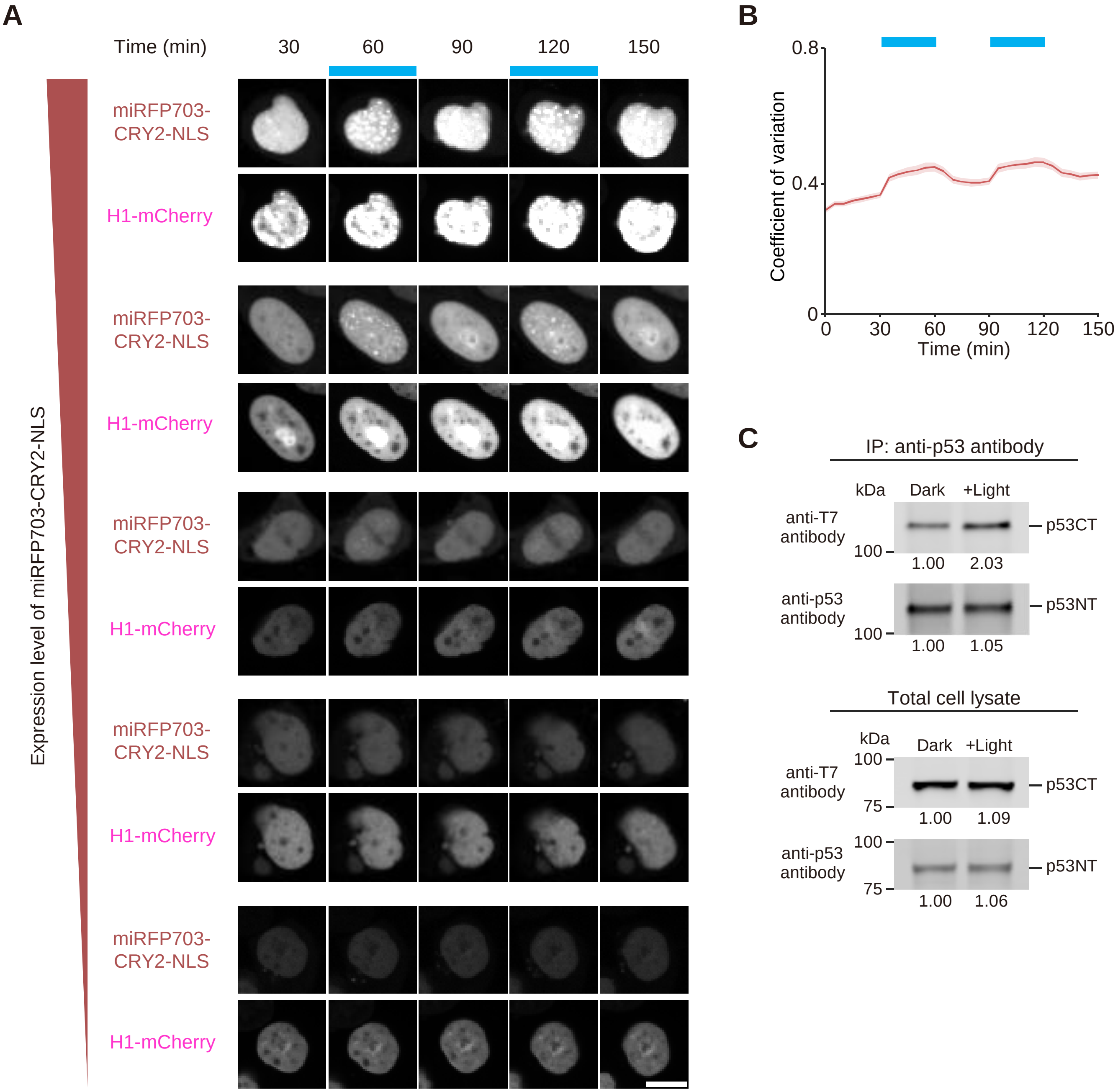

Figure S1. Characterization of CRY2 homo-oligomerization and CRY2-CIBN heterodimerization.

A. Light-dependent changes in subcellular localization of miRFP703-CRY-NLS fragments with different expression levels. The blue boxes indicate the time points at which blue light illumination was applied. H1-mCherry is a nuclear marker. Scale bar, 10 μm.

B. Temporal changes in the coefficient of variation of nuclear miRFP703 fluorescence intensity in cells expressing miRFP703-CRY-NLS. The plot shows the mean ± s.e.m. n = 91 cells.

C. HCT116 cells transiently expressing p53NT actuator and p53CT localizer were cultured under dark or blue light conditions for 15 min. After that, the cells were lysed, and their cell lysates were immuno-precipitated with monoclonal anti-p53 antibody, which recognized an epitope located in p53NT. The precipted samples (upper) and total cell lysates (lower) were subjected to western blotting with anti-T7 antibody and anti-p53 antibody. The representative results are shown.

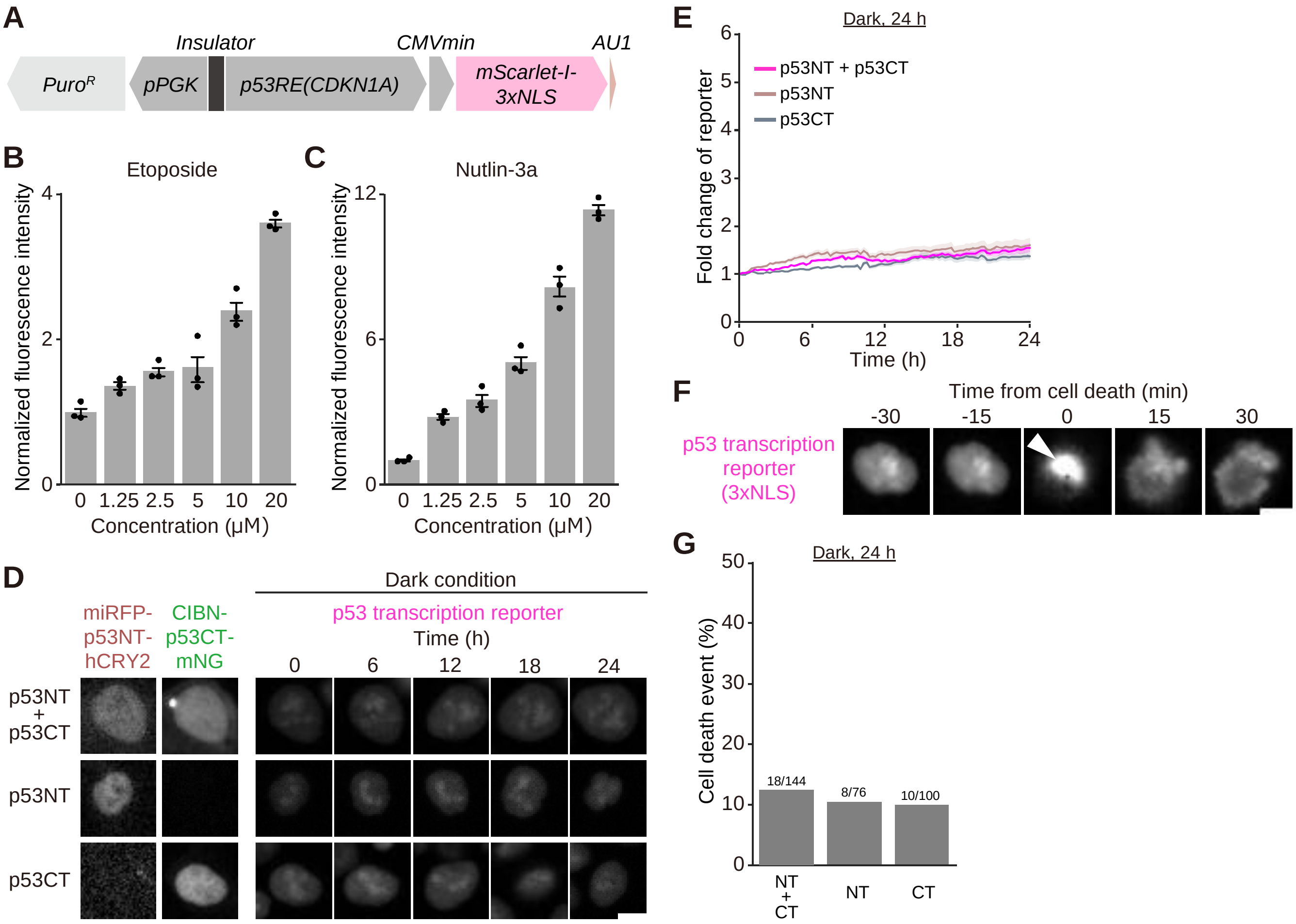

Figure S2. Functional characterization of the p53 transcriptional reporter and behavior of Opto-p53 under dark conditions.

A. Schematic representation of the reporter system used in this study. Puro^R, Puromycin resistance gene; pPGK, phosphoglycerate kinase promoter; CMVmin, CMV minimal promoter; AU1, AU-rich element.

B. Dose-response of the stable cell line harboring p53 transcription reporter 24 hours after treatment with the indicated concentration of etoposide.

C. Dose-response of the stable cell line harboring p53 transcription reporter 24 hours after treatment with the indicated concentration of nutlin-3a.

D. Expression patterns of Opto-p53 actuator or localizer and changes in p53 transcription reporter under dark conditions. Scale bar, 10 μm.

E. Fold changes in the p53 transcription reporter under dark conditions. The plot shows the mean ± s.e.m. p53NT + p53CT, n = 144 cells; p53NT, n = 76 cells; p53CT, n = 100 cells.

F. Morphological changes in cell death. White arrowheads indicate chromatin compaction associated with cell death. Cell images were obtained every 15 minutes.

G. Quantification of cell death in each condition. The numbers on each grey bar indicate the number of dead cells/total cells. *P*-values are as follows. NT+CT vs NT, 1.0; NT+CT vs CT, 1.0; NT vs CT, 1.0. Statistical analysis was performed using the chi-squared test with Bonferroni correction.

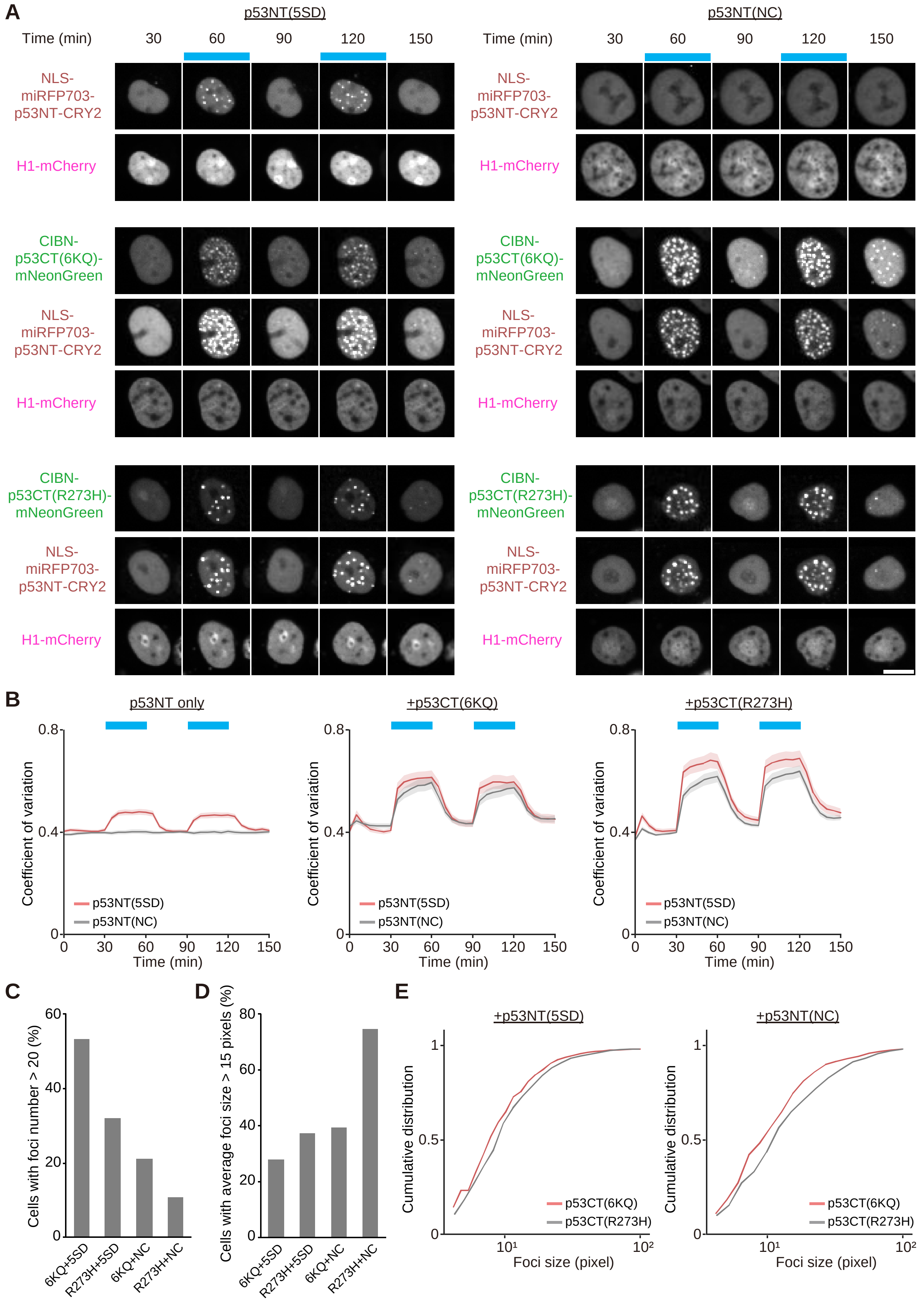
A
p53NT(5SD)
p53NT(NC)
Time (min)
30
60
90
120
150
NLS-miRFP703-p53NT-CRY2
H1-mCherry
CIBN-p53CT(6KQ)-mNeonGreen
CIBN-p53CT(R273H)-mNeonGreen
B
p53NT only
+p53CT(6KQ)
+p53CT(R273H)
Coefficient of variation
p53NT(5SD)
p53NT(NC)
Time (min)
C
Cells with foci number > 20 (%)
6KQ+5SD
R273H+5SD
6KQ+NC
R273H+NC
D
Cells with average foci size > 15 pixels (%)
E
+p53NT(5SD)
+p53NT(NC)
Cumulative distribution
p53CT(6KQ)
p53CT(R273H)
Foci size (pixel)

Figure S3. Subcellular localization changes in Opto-p53 fragments with mutations in the p53 domain.

A. Light-dependent changes in subcellular localization of each Opto-p53 actuator or localizer with different mutations in the p53 fragment. The blue boxes indicate the time points at which blue light illumination was applied. H1-mCherry is a nuclear marker. Scale bar, 10 µm. Upper panels, p53NT alone; middle panels, p53CT(6KQ) mutant + p53NT; lower panels, p53CT(R273H) mutant.
B. Temporal changes in the coefficient of variation of nuclear miRFP703 fluorescence intensity for each condition as shown in Figure S2A. Left panel, p53NT alone; middle panel, p53CT(6KQ) mutant + p53NT; right panel, p53CT(R273H) mutant. The plot shows the mean ± s.e.m. p53NT(5SD), n = 127 cells; p53NT(NC), n = 120 cells; p53CT(6KQ) + p53NT(5SD), n = 122 cells; p53CT(6KQ) + p53NT(NC), n = 151 cells; p53CT(R273H) + p53NT(5SD), n = 118 cells; p53CT(R273H) + p53NT(NC), n = 159 cells.
C. Cell population with the foci number > 20 in each co-expression condition.
D. Cell population with the average foci size > 15 pixels in each condition.
E. Cumulative distribution of foci size in all detected foci. Left panel, p53CT(6KQ) mutant + p53NT; right panel, p53CT(R273H) mutant. *p*-value: 3.0×10^{-6} for p53CT(6KQ) + p53NT(5SD) vs p53CT(6KQ) + p53NT(NC), 5.1×10^{-8} for p53CT(R273H) + p53NT(5SD) vs p53CT(R273H) + p53NT(NC). *p*-values were calculated by a Mann-Whitney U test.

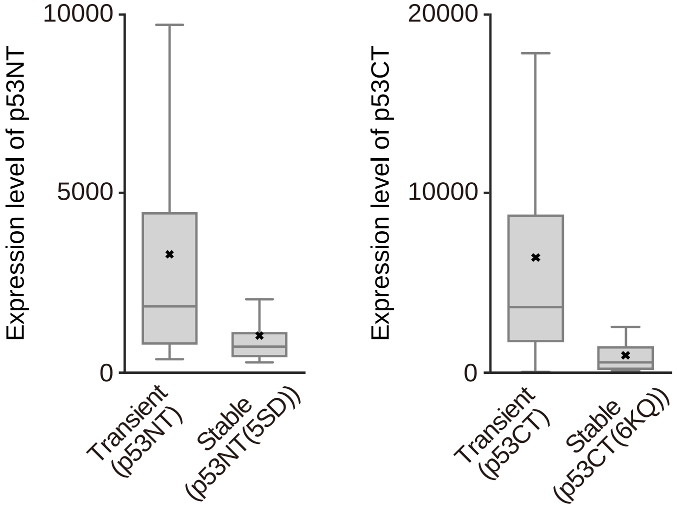

Figure S4. The comparison of the expression levels of p53NT and p53CT fragments.

The expression levels of Opto-p53 actuators (p53NT and p53NT(5SD)) (left) and Opto-p53 localizer (p53CT and p53CT(6KQ)) (right) are plotted as a box plot. Of note, the fluorescence intensities of these proteins were normalized by dividing by their exposure time. Horizontal lines and crosses indicate the medians and means of distribution, respectively. Boxes and whiskers include the values between the 25th and 75th percentiles or between the maximum and minimum values excluding outliers, respectively.

Table S1

Plasmid name	Figures	Source or reference	Sequence
pCSIIbleo-H1-mCherry	2, 3, S1, S3	This study	https://benchling.com/s/seq-8w1sVGlEYvw4uyexxMKw?m=slm-gKtz0EOAkyiXOqhoKjtM
pPBbsr2-miRFP703-dSal-p53NT(1-97)-S15D-hCRY2-NLS	2, 3, S2	This study	https://benchling.com/s/seq-iaymBMZnxtC3UG3iM9Kj?m=slm-Ki8MNcdXMSnSiICJc5WF
pPBbsr2-miRFP703-dSal-VP16minADx3-hCRY2-NLS	2, 3	This study	https://benchling.com/s/seq-Z27cmjXf0ZOWdL9Q0ZE7?m=slm-tU3F0ok9Pb2DYIy0k3ZJ
pCAGGS-CIBN-p53CT(98-393)-mNeonGreen	2, 3, S2	This study	https://benchling.com/s/seq-SmXmLCJPBWd2Ezilwum3?m=slm-V9iSpDwed2bFAJ3BnxFV
pPB-p53RE(CDKN1A)-CMVmin-mScarlet-I-NLSx3-AU1-PGKpuro	3, 4, S2	This study	https://benchling.com/s/seq-lv1G3m5olUoQKYBtBMca?m=slm-CbOooCTkrhJDY4fPlsMi
pPBbsr2-NLSx3-miRFP703-dSal-p53NT(1-97)-5SD-lin-hCRY2	4	This study	https://benchling.com/s/seq-fZiB5aeNnaWCmscug3kT?m=slm-gPuQW5SitzBdnclEQHhi
pPBbsr2-NLSx3-miRFP703-dSal-p53NT(1-97)-L22Q/W23S/W53Q/F54S-lin-hCRY2	4	This study	https://benchling.com/s/seq-m6oqqZOadMHkaQ9eZi2E?m=slm-bQNOzruUFTAGNoZLV7Hl
pPBneo-CIBN-lin-p53CT(98-393)-6KQ-mNeonGreen-NLSx3	4	This study	https://benchling.com/s/seq-xlq0aODx512OPaNJg2Kh?m=slm-SrXB4VYMFipwHP2l6PkD
pPBneo-CIBN-lin-p53CT(98-393)-R273H-mNeonGreen-NLSx3	4	This study	https://benchling.com/s/seq-R0dnqxz6dlOdlaldc5Yb?m=slm-UYRM4hZLBiayM7JI2GFL
pPBbsr2-miRFP703-dSal-lin-hCRY2-NLS	S1	This study	https://benchling.com/s/seq-lMmo585RZDRqqFKmtzxf?m=slm-pwb8jrlqveI9pc8yBtYS
pPBbsr2-miRFP703-dSal-p53NT(1-97)-hCRY2-NLS	S1	This study	https://benchling.com/s/seq-EpOd8JhGCKmY4UjV4AUe?m=slm-QIOZtokIsfk4jowiGubE
pCAGGS-CIBN-p53CT(98-393)-mNeonGreen-T7	S1	This study	https://benchling.com/s/seq-E7m07OfKFjwFSPOabgdR?m=slm-ggtiaM9QDfYNQHAPLf3U
pPBbsr2-SuperNLS-miRFP703-dSal-p53NT(1-97)-5SD-lin-hCRY2	S3	This study	https://benchling.com/s/seq-WMn7te2KkdVCZ6pTtptT?m=slm-qHednjcL97CTyZZk3pWC

pPBbsr2-SuperNLS-miRFP703-dSal-p53NT(1-97)-L22Q/W23S/W53Q/F54S-lin-hCRY2	S3	This study	https://benchling.com/s/seq-1eUakm7jJFRSaz6FaAt3?m=slm-ewWwpMXkDH3YEo2u0WmH
pPBneo-CIBN-lin-p53CT(98-393)-6KQ-mNeonGreen-SuperNLS	S3	This study	https://benchling.com/s/seq-T3aCEpiu08SmIh1NcjCh?m=slm-xlLyqvUYU2a9LxdKieqJ
pPBneo-CIBN-lin-p53CT(98-393)-R273H-mNeonGreen-SuperNLS	S3	This study	https://benchling.com/s/seq-S6yezfzJ0OD1iqbU2U0a?m=slm-ZpfYeI4bB5vzkFqNAyaw

Table S1. Plasmid list used in this study.

Movie 1

HCT116 cells transiently expressing the p53NT actuator were cultured on a 4-well glass-bottom dish. Time-lapse imaging was performed using a spinning disk confocal microscope. The cells were repeatedly illuminated with blue light at 30 min intervals. Images were acquired every 5 min. Total imaging time = 150 min.

Movie 2

HCT116 cells harboring a p53 transcriptional reporter were cultured on a 4-well glass-bottom dish and transiently expressing Opto-p53. Time-lapse imaging was performed using a wide-field microscope. The cells were continuously illuminated with blue light at 24 hours. Images were acquired every 15 min. Total imaging time = 24 hours.
